# Heat stress reduces sexual development and affects pathogenesis of *Eimeria maxima* in meat-type chickens

**DOI:** 10.1038/s41598-020-67330-w

**Published:** 2020-07-01

**Authors:** Gustavo H. Schneiders, James C. Foutz, Marie C. Milfort, Ahmed F. A. Ghareeb, Alberta L. Fuller, Romdhane Rekaya, Susan M. Williams, Samuel E. Aggrey

**Affiliations:** 1 NutriGenomics Laboratory, Department of Poultry Science, University of Georgia, Athens, GA 30602 USA; 2 Animal and Dairy Science, University of Georgia, Athens, GA 30602 USA; 3 Institute of Bioinformatics, University of Georgia, Athens, GA 30602 USA; 4 Poultry Diagnostic and Research Center, University of Georgia, Athens, GA 30602 USA

**Keywords:** Developmental biology, Disease model

## Abstract

Coccidiosis, caused by *Eimeria* spp. presents a self-limiting intestinal infection of poultry. Intestinal replication of the parasite causes severe morphological alterations to the host gastrointestinal tract, marked, among others, by the disruption of the intestinal barrier. We have previously reported a significant reduction in merozoite replication and oocyst shedding in *E. tenella *in vitro and in vivo. The objective of this study was to investigate the pathogenesis of *E*. *maxima* infection in broiler chickens under heat stress (HS) and mRNA expression of host cytokines that might affect the curtailed development of the parasite. We herein demonstrate that there is a significant detrimental effect of HS on the pathogenesis of *E*. *maxima* infection in broilers. There was a restricted replication of the parasite in HS chickens evidenced by significantly reduced oocyst shedding and disruption of the intestinal blood barrier. Gene expression of parasite genes demonstrated curtailed sexual reproduction of *E*. *maxima* in HS chickens. There was downregulation of *Eimeria* spp. genes related to gamete fusion, oocyst shedding, mitosis and spermiogenesis. Host gene expression indicates alterations in the cytokine expression that could be related to reduced parasite development in vivo.

## Introduction

*Eimeria* spp*.* is an apicomplexan parasite, the causative agent of coccidiosis, a disease of high economic impact in poultry production worldwide. The parasite’s life-cycle is comprised of several cycles of endogenous asexual replication followed by sexual development that results in the formation of the oocysts, later excreted in the feces^1^. *Eimeria* (*E*.) *maxima* is one of the seven recognized species of coccidia that infect the chicken. The disease is marked by reduced growth, apathy, diarrhea and in severe cases, mortality. Clinical signs often include emaciation, pallor, roughening of feathers and anorexia. Abundance of yellow–orange mucus and fluid in the distal portion of the jejunum and proximal portion of the ileum, edema, thickening and disruption of the mucosa and sometimes presence of blood in the intestinal lumen are observed at necropsy^2^.

Heat stress (HS) is one of the major environmental problems of poultry production in tropical and subtropical regions. Stress is a predisposing factor of immunosuppression in broilers, offering a good opportunity to normal commensals to induce infection and disease^3–5^. Heat stress has been reported to enhance pathogen attachment, colonization, shedding, reduce intestinal crypt depth and impact food safety risks^5–8^. The increase in pathogen colonization in heat stressed chickens is believed to be related to the disturbances in microbiota composition, thereby leading to a loss of protection against pathogenic microorganisms^8^.

Contrary to the detrimental effects of HS in the outcome of infection with most poultry pathogens, we have previously demonstrated that the increase in 2 °C in the temperature of incubation of *E. tenella* significantly reduces asexual replication in vitro and that HS significantly reduces the outcome of *E. tenella* infection in broilers, as marked by reduction in merozoite production and oocyst shedding^9^. Similarly, HS also significantly reduces *E*. *acervulina* oocyst shedding in broilers^10^. It remains unclear how HS curtails *Eimeria* spp. replication in vivo.

Thus, the objective of this study was to investigate the effect of HS on the pathogenesis of *E*. *maxima* infection in broilers, as well as differential expression of host cytokines that might affect the curtailed development of the parasite. Together, these data indicate that HS of the host significantly reduces the sexual stages of *E*. *maxima *in vivo. Moreover, we provide information on the relevance of HS control in the host response of birds not only for animal welfare and health, but also for their response to pathogens.

## Results

### Coccidiosis and HS cause decreased production parameters

**Figure 1:**
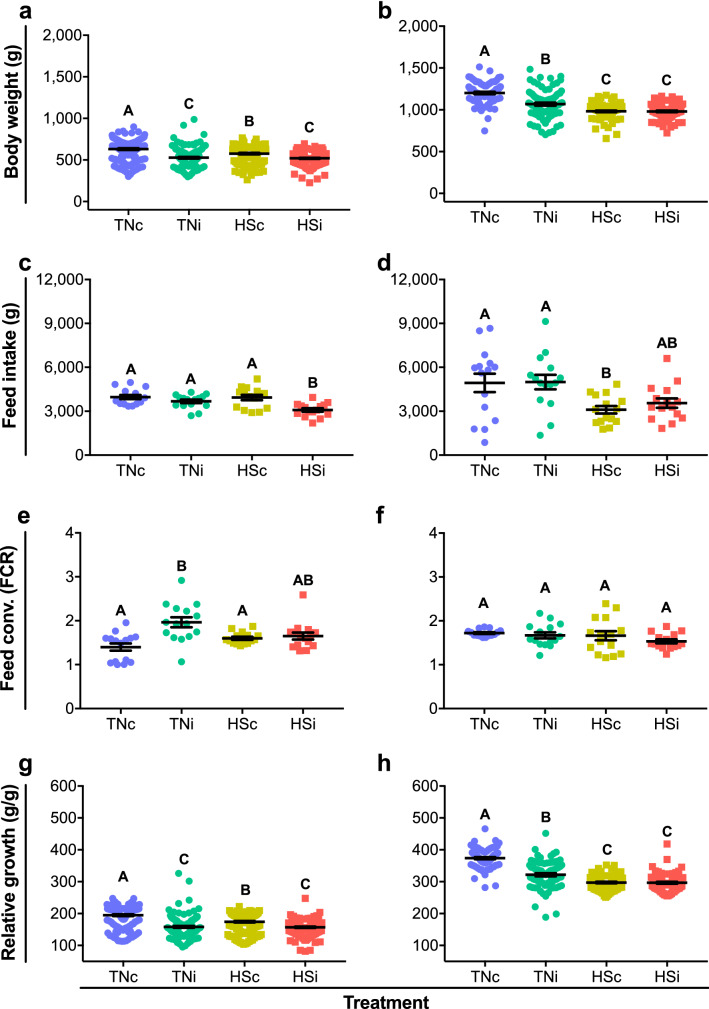
Body weight at 7 (**a**) and 14 dpi (**b**); feed intake at 7 (**c**) and 14 dpi (**d**), feed conversion ratio (FCR) at 7 (**e**) and 14 dpi (**f**), and relative growth at 7 (**g**) and 14 dpi (**h**). Control chickens were mock infected and housed at thermoneutral (TNc) or heat stress (HSc) environments. 200.000 sporulated oocysts of *E*. *maxima* were given via gavage to TNi and HSi chickens. Mean and standard error of the mean (SEM) are depicted. Normal data was analyzed by one-way-ANOVA, non-parametric data was analyzed by Kruskal–Wallis. All tests were performed at 5% level of significance (p < 0.05). Significant differences between the groups are indicated by different superscript letters.

The effects of HS and *E*. *maxima* infection in BW at 7 dpi (Fig. 1a) and 14 dpi (Fig. 1b) are depicted. At 7 dpi, the HSc chickens had lower BW (576 ± 103 g) as compared to TNc (631 ± 127 g; p < 0.0011). The HSi group had lower BW (520 ± 81 g) as compared to TNc and HSc (p < 0.0001), however not statistically different from TNi (p = 0.9344). At 14 dpi, TNi chickens had lower BW (1,068 ± 169 g) as compared to TNc (1,201 ± 125 g; p < 0.0001), however still higher as compared to HSc (982 ± 91 g; p < 0.0001) and HSi (981 ± 90 g; p < 0.0001). While no statistical differences were observed in BW between HSc and HSi at 14 dpi (p > 0.9999), both treatments presented significantly lower BW as compared to TNi (p < 0.0001).

Feed intake at 7 and 14 dpi is shown in Fig. 1c, d, respectively. The TNc group of chickens had feed intake at 7 dpi (2,900 ± 220 g) higher as compared to TNi (1,671 ± 121 g; p < 0.0001), HSc (1,931 ± 181 g; p = 0.0007) and HSi (1,087 ± 120 g; p < 0.0001). Chickens from the TNi group had feed intake similar to HSc (p = 0.6863) and HSi (p = 0.0729), however, HSc chickens showed feed intake at 7 dpi higher as compared to HSi (p = 0.0037). At 14 dpi, TNc chickens had an average feed intake (4,931 ± 630 g) similar to TNi (4,926 ± 544 g; p > 0.9999) and HSi (3,486 ± 351 g; p = 0.1457), and higher as compared to HSc (2,169 ± 272 g; p = 0.0007). Similarly, feed intake was similar between TNi and HSi (p = 0.1477), however higher in TNi as compared to HSc (p = 0.0007). No differences in feed intake were observed between HSc chickens as compared to HSi (p = 0.3202).

Feed conversion ratio (FCR) at 7 dpi and 14 dpi are presented in Fig. 1e, f, respectively. At 7 dpi, chickens from the TNc group had FCR of 1.40 ± 0.08, statistically similar to HSc (1.6 ± 0.03; p = 0.5597) and HSi (1.65 ± 0.08; p = 0.2194), however statistically lower as compared to TNi (1.97 ± 0.11; p < 0.0001). TNi chickens had statistically higher FCR as compared to HSc (p = 0.0169), however statistically similar to HSi (p = 0.0563). No statistical differences were detected in FCR between HSc and HSi at 7 dpi (p > 0.9999). At 14 dpi, FCR ranged between 1.53 ± 0.05 and 1.72 ± 0.02, with no statistically significant differences between the groups (p ≥ 0.3133).

Relative growth (RG) at 7 and 14 dpi is presented in Fig. 1g, h, respectively. At 7 dpi, the average RG for TNc chickens (195 ± 2.795) was higher when compared to TNi (158 ± 2.668; p < 0.0001), HSc (174 ± 2.283; p < 0.0001) and HSi (157 ± 1.951; p < 0.0001). HSc had RG higher as compared to TNi and HSi (p < 0.0001). No differences were observed in RG between TNi and HSi (p > 0.9999) at 7 dpi. Relative growth at 14 dpi was significantly higher in TNc (374 ± 3.240) when compared with TNi (322 ± 4.635; p < 0.0001), HSc (298 ± 2.402; p < 0.0001) and HSi (297 ± 3.119; p < 0.0001). Similarly, TNi had RG statistically higher as compared to HSc (p = 0.0002) and HSi (p < 0.0001). No differences in RG were observed between HSc and HSi at 14 dpi (p > 0.9999).

### *E*. *maxima*-specific intestinal lesions

**Figure 2:**
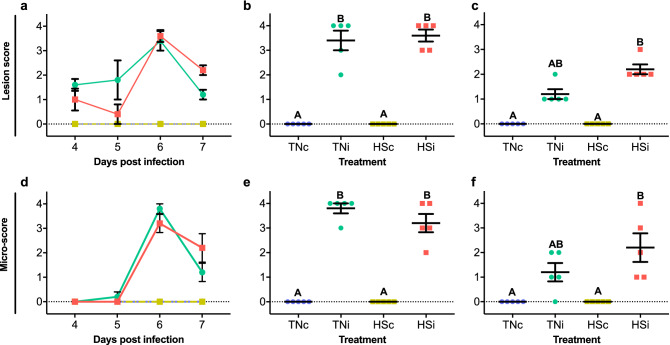
Lesion scores from 4 to 7 dpi (**a**), at 6 dpi (**b**) and 7 dpi (**c**). Microscopic scores from 4 to 7 dpi (**d**), at 6 dpi (**e**) and 7 dpi (**f**). of chickens infected with *E. maxima* and housed at thermoneutral (TNi; green) or heat stress (HSi; red) environment, as compared to uninfected chickens housed at thermoneutral (TNc; blue) or heat stress (HSc; yellow) environments, assessed from 4 to 6 dpi. Mean and standard error of the mean (SEM) are depicted. Data were analyzed by Kruskal–Wallis test. All tests were performed at 5% level of significance (p < 0.05). Significant differences between the groups are indicated by different superscript letters.

The peak of intestinal gross lesions was at 6 dpi (Fig. 2a), in which lesion scores are significantly higher (p < 0.0183) in TNi (3.4 ± 0.40) and HSi (3.6 ± 0.24) chickens as compared to zero recorded in the controls (Fig. 2b). There were no differences between lesion scores from TNi and HSi (p > 0.9999). At 7 dpi (Fig. 2c), lesion scores in TNi (1.2 ± 0.20) were similar to the control groups (zero; p = 0.1337). Although HSi (2.2 ± 0.20) was statistically similar to TNi (p > 0.9999), scores recorded for this group were still significantly higher as compared to the controls. Similarly, the highest microscores was recorded at 6 dpi (Fig. 2d). Microscores at 6 dpi (Fig. 2e) were similar between TNi (3.8 ± 0.20) and HSi (3.2 ± 0.37; p > 0.9999), both significantly higher as compared to zero (p ≤ 0.05). At 7 dpi (Fig. 2f), microscores recorded for TNi (1.2 ± 0.37) were statistically similar to zero, recorded from TNc and HSc (p = 0.1761) or to HSi (2.2 ± 0.58; p > 0.9999), however, scores recorded for HSi were higher as compared to TNc and HSc (p = 0.0108).

### Disruption of the intestinal blood barrier precedes oocyst shedding

**Figure 3:**
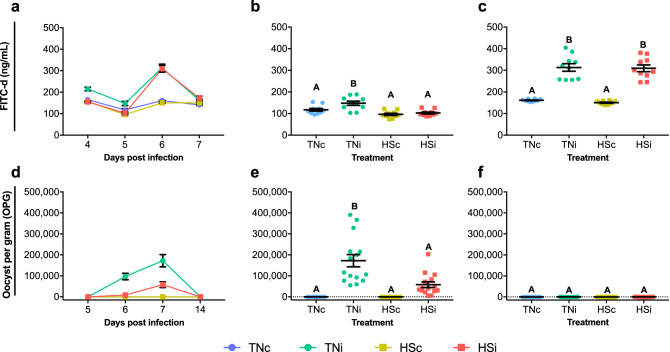
Quantification of fluorescein isothiocyanate dextran (FITC-d) in serum samples collected from 4 to 7 dpi (**a**), at 5 dpi (**b**) and 6 dpi (**c**). Oocyst shedding from 5, 6, 7 and 14 dpi (**d**), at 7 dpi (**e**) and 14 dpi (**f**) of chickens infected with *E. maxima* and housed at thermoneutral (TNi; green) or heat stress (HSi; red) environment, as compared to uninfected chickens housed at thermoneutral (TNc; blue) or heat stress (HSc; yellow) environments. Mean and standard error of the mean (SEM) are depicted. Normal data was analyzed by one-way-ANOVA, non-parametric data was analyzed by Kruskal–Wallis. All tests were performed at 5% level of significance (p < 0.05). Significant differences between the groups are indicated by different superscript letters.

The peak of intestinal barrier disruption, as measured by FITC-d (ng/mL) translocation from the lumen to the serum, was at 6 dpi (Fig. 3a). At 5 dpi (Fig. 3b), the TNc group showed FITC-d concentration (117 ± 6.39) similar to HSc (96 ± 5.08; p = 0.1929) and HSi (103 ± 4.48; p = 0.7990), however, the TNi (148 ± 9.77) group higher FITC-d concentration compared to TNc (p = 0.0184), HSc (p < 0.0001) and HSi (p = 0.0002). At 6 dpi (Fig. 3c), FITC-d concentration was higher (p < 0.0001) in TNi (313 ± 17.58) and HSi (309 ± 15.75) chickens as compared to TNc (161 ± 1.82) and HSc (150 ± 2.44). No significant differences were detected between TNc and HSc (p > 0.9999) or between TNi and HSi (p > 0.9999).

### Heat stress limits *E. maxima* replication and oocyst shedding

**Figure 4:**
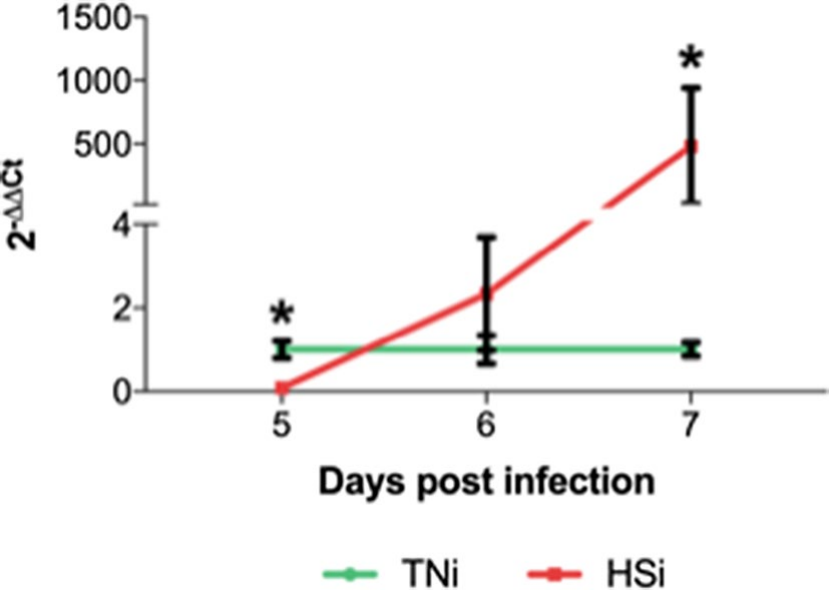
Quantification of *Eimeria maxima* mRNA in ileum samples of chickens infected with *E. maxima* and housed at heat stress (HSi; red) environment as compared to infected chickens housed at thermoneutral (TNi; green) environment, assessed by RT-qPCR from 5 to 7 dpi and expressed as 2^−∆∆Ct^. Mean and standard error of the mean (SEM) are depicted. Data were analyzed by GLM procedure with multiple comparisons corrected with Tukey’s method at 5% level of significance (p < 0.05). Significant differences between the groups within the same day are indicated by the superscript asterisk.

The peak of oocyst shedding was at 7 dpi (Fig. 3d). While no oocysts were shed from chickens from TNc and HSc groups, oocyst shedding at the HSi group averaged 58,051 ± 13,45 OPG at 7 dpi (Fig. 3e), demonstrating a tendency to be higher as compared to zero (p = 0.0795). Oocyst shedding averaged 172,429 ± 29,13 OPG for TNi chickens, higher as compared to HSi, and the controls TNc and HSc (zero; p < 0.0001). Minimal oocyst shedding was detected at 14 dpi (Fig. 3f), with averages 129 ± 55.02 and 133 ± 63.09 for TNi and HSi, respectively, however these were not statistically different from zero (p ≥ 0.1689). The presence of *E*. *maxima* RNA in ileum samples was detected by RT-qPCR at from 5 to 7 dpi with results, expressed as 2^−∆∆*Ct*^, depicted in Fig. 4. At 5 dpi, HSi chickens showed significantly lower expression of 18 s (0.08 ± 0.02) as compared to TNc (1 ± 0.20; p < 0.05). At 6 dpi the expression of 18 s was higher in HSi (2.33 ± 1.36), however, this was not statistically different from TNi (1 ± 0.34; P ≥ 0.05). At 7 dpi, there was a significantly higher quantification of 18 s in HSi (482 ± 458) chickens as compared to TNi (1 ± 0.16; p < 0.05). There was no detection of 18 s in samples from TNc and HSc.

**Figure 5:**
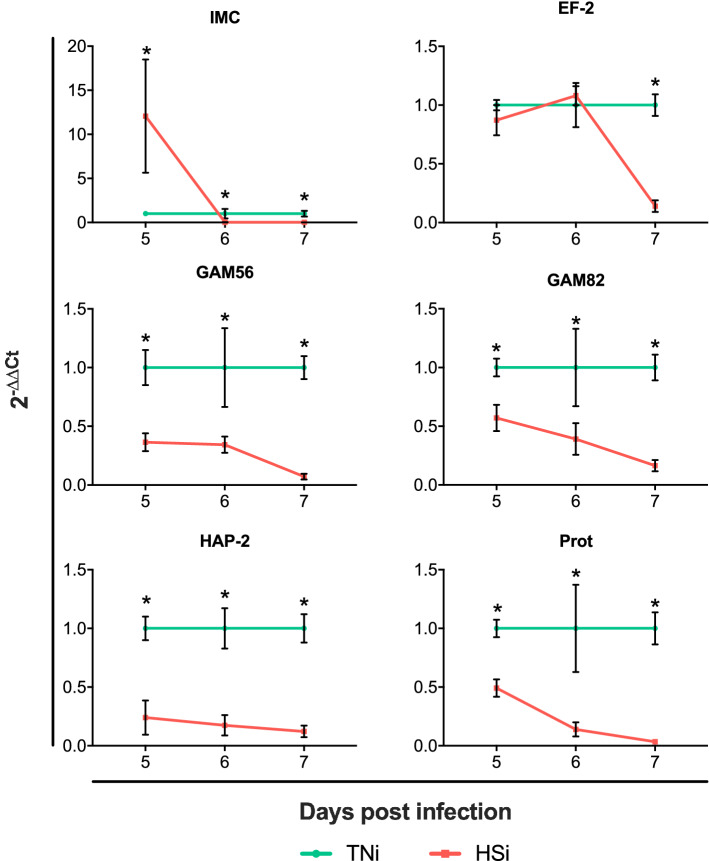
*Eimeria* spp. gene expression analysis in ileum samples collected from chickens infected with *E. maxima* and housed at heat stress (HSi; red) environment as compared to infected chickens housed at thermoneutral (TNi; green) environment, assessed by RT-qPCR from 5 to 7 dpi and expressed as 2^−∆∆Ct^. Genes analyzed were inner membrane complex 1 (IMC), elongation factor 2 (EF-2), gametocyte protein 56 (GAM56), gametocyte protein 82 (GAM82), hapless 2 (HAP-2) and protamine (Prot). Data were analyzed by GLM procedure with multiple comparisons corrected with Tukey’s method at 5% level of significance (p < 0.05). Mean and standard error of the mean (SEM) are depicted. Significant differences between the groups within the same day are indicated by the superscript asterisk.

The development of *E*. *maxima* in the small intestine was assessed by quantification of the expression of *E*. *maxima*-specific genes (mRNA) in ileum sections at 5, 6 and 7 dpi, with results depicted on Fig. 5 as 2^−∆Ct^. Genes analyzed were inner membrane complex 1 (IMC), elongation factor 2 (EF-2), expressed throughout the development of the parasite; gametocyte protein 56 (GAM56), gametocyte protein 82 (GAM82), related to macrogametocyte development; hapless 2 (HAP-2) and protamine (Prot) related to microgametocyte development. There was no detection of *Eimeria*-specific gene expression in samples from TNc and HSc. At 5 dpi, IMC was significantly upregulated in HSi (12.06 ± 6.42 vs 1 ± 0.06), followed by significant downregulation at 6 (0.02 ± 0.02 vs 1 ± 0.55) and 7 (0.02 ± 0.01 vs 1 ± 0.33) dpi as compared to TNi. The expression of EF-2 was similar between HSi and TNi at 5 (0.87 ± 0.13 vs 1 ± 0.04) and 6 dpi (1.08 ± 0.08 vs 1 ± 0.19), however, HSi showed significant downregulation of EF-2 at 7 dpi (0.14 ± 0.05 vs 1 ± 0.09). The expression of GAM56 was significantly downregulated in HSi at 5 (0.36 ± 0.08 vs 1 ± 0.15), 6 (0.34 ± 0.07 vs 1 ± 0.34) and 7 dpi (0.07 ± 0.02 vs 1 ± 0.10). The expression of GAM82 was significantly downregulated in HSi at 5 (0.57 ± 0.11 vs 1 ± 0.08), 6 (0.39 ± 0.13 vs 1 ± 0.33) and 7 dpi (0.16 ± 0.05 vs 1 ± 0.11). The expression of HAP-2 was significantly downregulated in HSi at 5 (0.24 ± 0.15 vs 1 ± 0.10), 6 (0.17 ± 0.09 vs 1 ± 0.17) and 7 dpi (0.12 ± 0.05 vs 1 ± 0.12). The expression of Prot was significantly downregulated in HSi at 5 (0.49 ± 0.07 vs 1 ± 0.07), 6 (0.14 ± 0.06 vs 1 ± 0.37) and 7 dpi (0.03 ± 0.02 vs 1 ± 0.14).

### *Eimeria maxima* detection by histopathology

**Figure 6:**
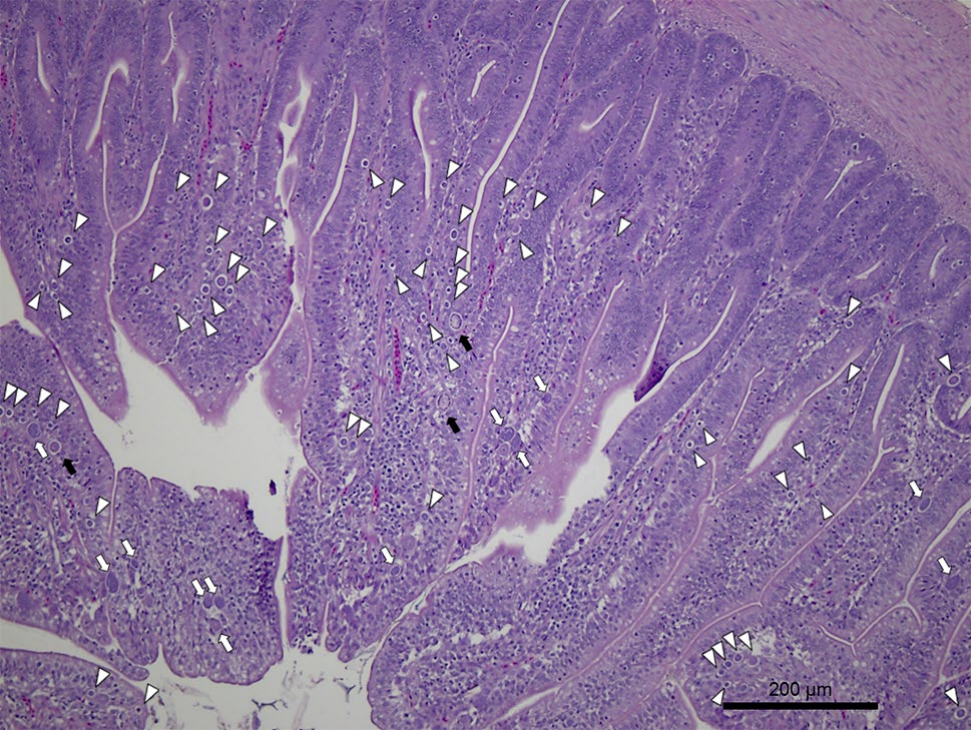
Photomicrographs of *E. maxima* in samples of ileum from TNi chickens. Three macrogametocytes (black arrows), 12 microgametocytes (white arrows) and 54 schizonts (white arrowheads) can be seen in the lamina propria at 7 dpi. Tissues were stained with hematoxylin and eosin (H&E).

**Figure 7:**
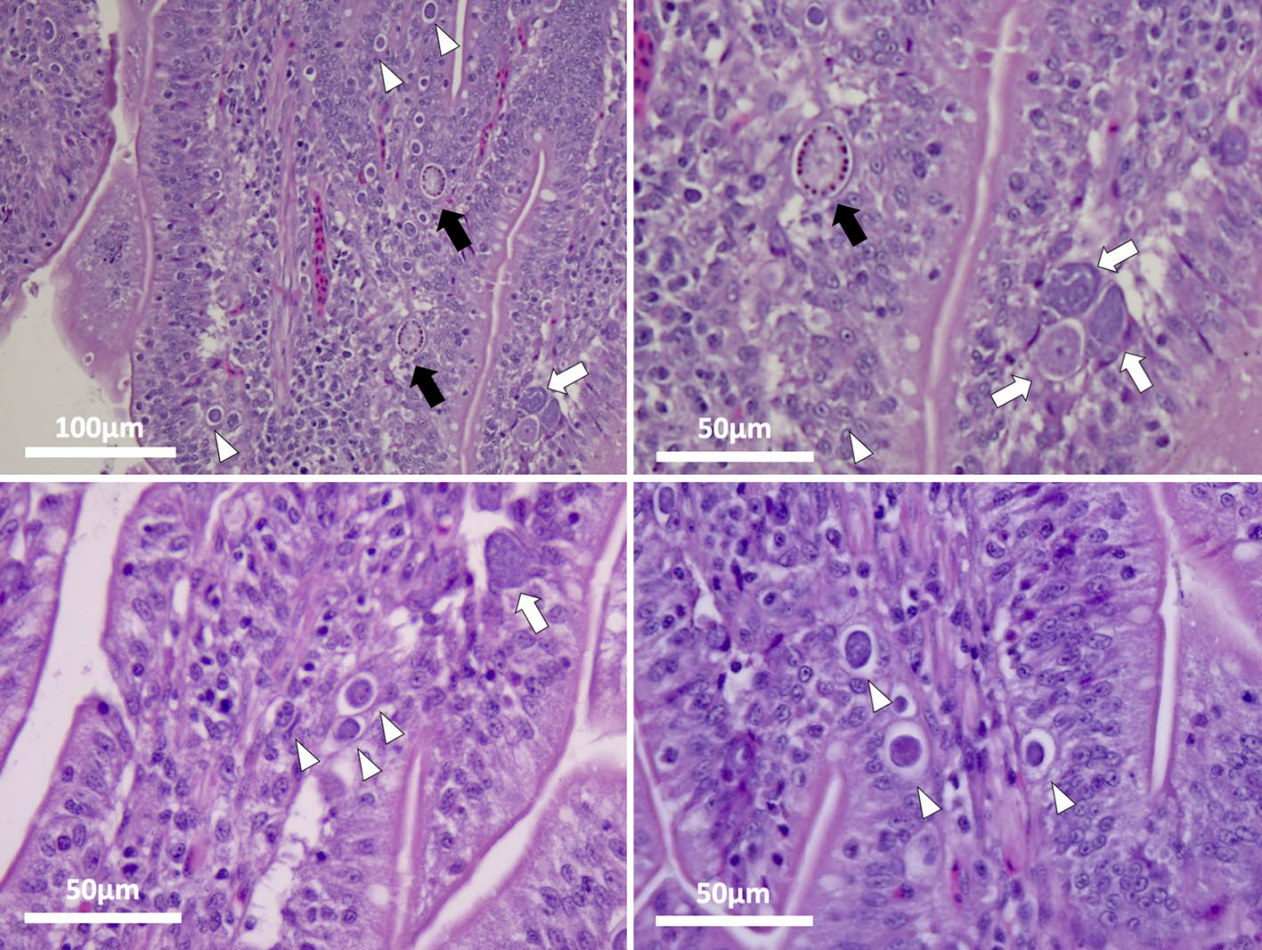
Photomicrographs of *E. maxima* in samples of ileum from TNi chickens. Macrogametocytes (black arrows), microgametocytes (white arrows) and schizonts (white arrowheads) can be seen in the lamina propria at 7 dpi. Tissues were stained with hematoxylin and eosin (H&E).

**Figure 8:**
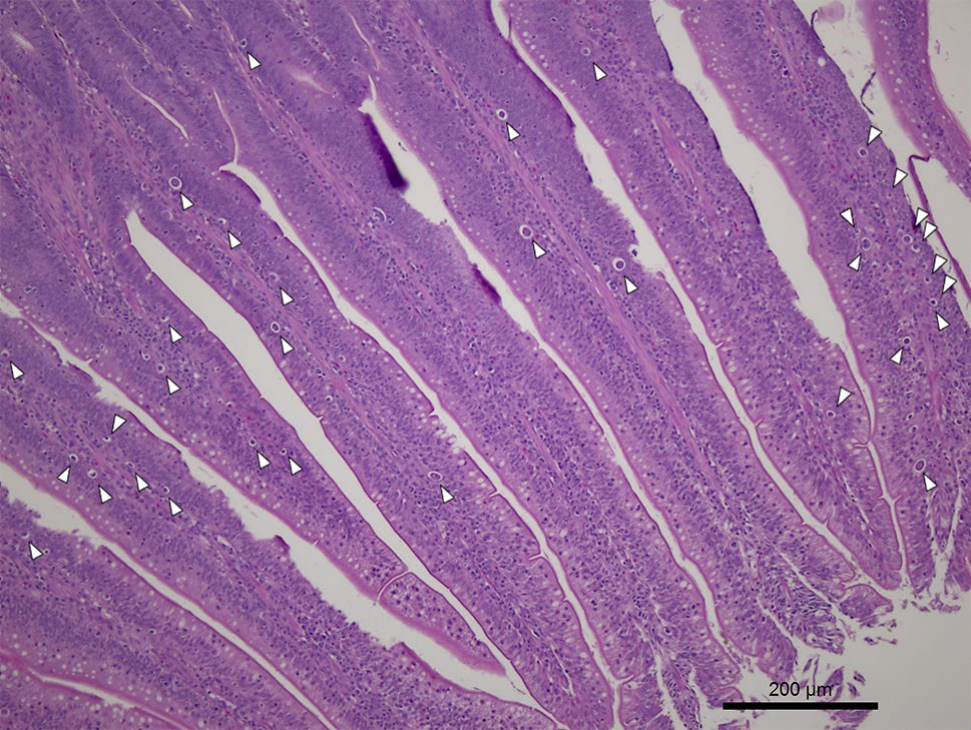
Photomicrographs of *E. maxima* in samples of ileum from HSi chickens. Thirty-three schizonts (white arrowheads) can be seen in the lamina propria at 7 dpi. Tissues were stained with hematoxylin and eosin (H&E).

**Figure 9:**
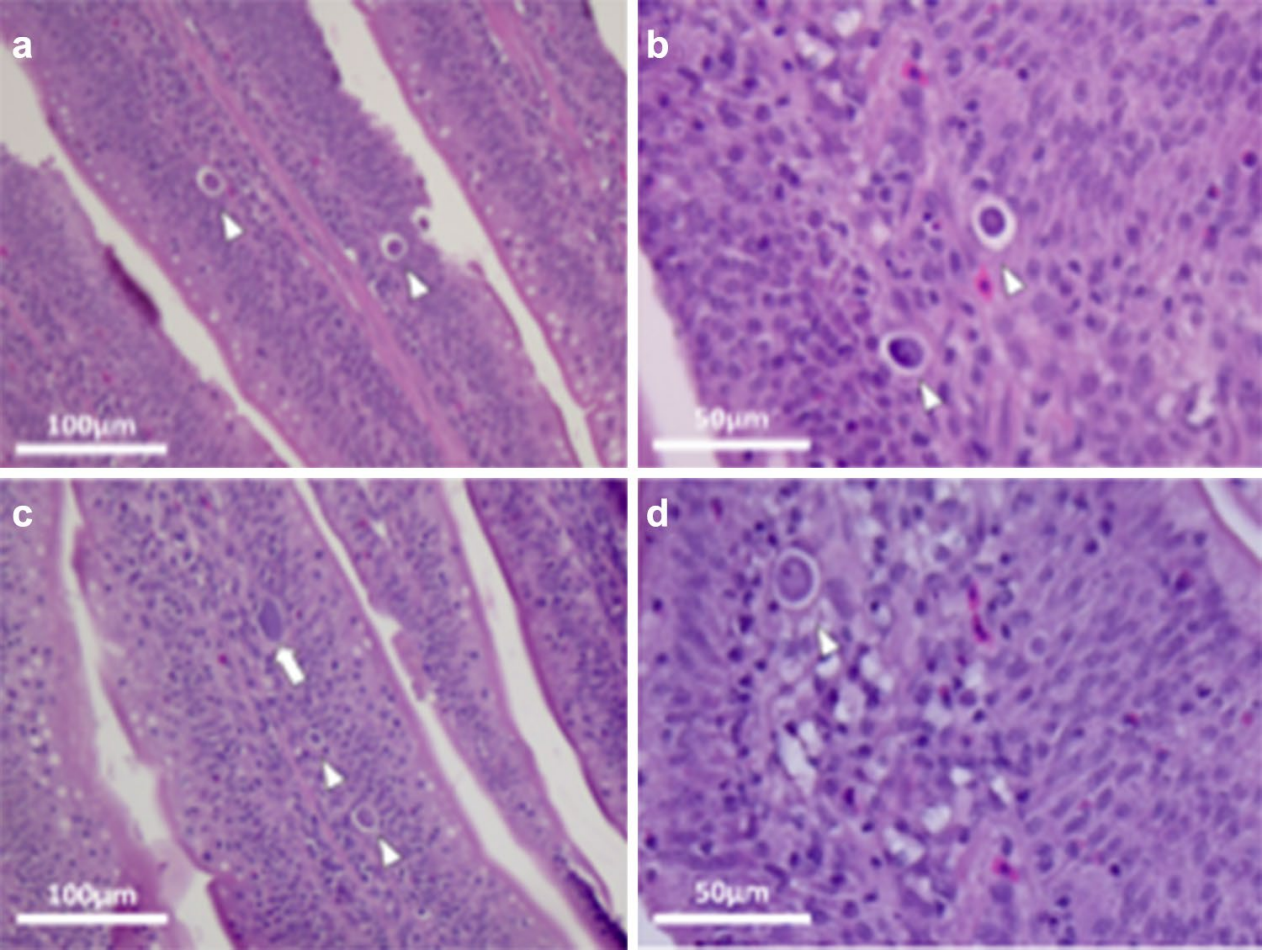
Photomicrographs of *E. maxima* in samples of ileum from HSi chickens. Schizonts (white arrowheads) and a single microgametocyte (white arrow) can be seen in the lamina propria at 7 dpi. Tissues were stained with hematoxylin and eosin (H&E).

There was no histological evidence of coccidia infection in sections from uninfected control chickens (TNc and HSc) throughout the experiment. Mild to severe coccidial enteritis was observed at 7 dpi in chickens from the TNi group (Fig. 6). The diagnosis was marked by the presence of *E*. *maxima* in several stages of development in the lamina propria and by high numbers of organisms present in each section of intestine, varying from asexual to sexual stages (Figs. 6, 7). On the other hand, chickens from the HSi group were diagnosed as rare to minimal coccidial enteritis (Fig. 8), marked by the presence of scattered schizonts in the lamina propria (Fig. 8) and rare sexual stages (Fig. 9).

### Host immunomodulation during HS and *E*. *maxima* infection

**Figure 10:**
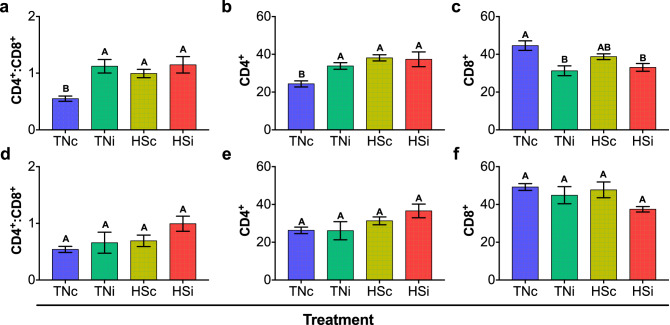
T lymphocyte cell populations and ratio in the spleen of chickens infected with *E. maxima* and housed at thermoneutral (TNi; green) or heat stress (HSi; red) environment, as compared to uninfected chickens housed at thermoneutral (TNc; blue) or heat stress (HSc; yellow) environments. CD4^+^:CD8^+^ ratio at 7 dpi (**a**), CD4^+^ percentage at 7 dpi (**b**), CD8^+^ percentage at 7 dpi (**c**), CD4^+^:CD8^+^ ratio at 14 dpi (**d**), CD4^+^ percentage at 14 dpi (**e**), CD8^+^ percentage at 14 dpi (**f**). Data were analyzed by GLM procedure with multiple comparisons corrected with Tukey’s method at 5% level of significance (p < 0.05). Mean and standard error of the mean (SEM) are depicted. Significant differences between the groups are indicated by different superscript letters.

CD4^+^:CD8^+^ ratio was calculated from the number of cells labelled with the fluorescent monoclonal antibodies anti-CD4 or anti-CD8 analyzed by flow cytometry. At 7 dpi (Fig. 10a), CD4^+^:CD8^+^ ratio was significantly higher in chickens from the treatments TNi (1.12 ± 0.12), HSc (0.99 ± 0.07) and HSi (1.15 ± 0.14) as compared to TNc (0.55 ± 0.05; p < 0.05). Similarly, there was a significantly lower percentage of CD4^+^ cells in TNc (24.35 ± 1.64) as compared to TNi (33.82 ± 1.74), HSc (38.14 ± 1.62) and HSi (37.38 ± 3.88; Fig. 10b). There was a significantly higher percentage of CD8^+^ cells in TNc (44.63 ± 2.53) as compared to TNi (31.28 ± 2.63) and HSi (33.08 ± 2.03), with no statistical difference between TNc and HSc, and between HSc, TNi and HSi (Fig. 10c). At 14 dpi, CD4^+^:CD8^+^ ratio ranged between 0.54 ± 0.05 and 0.99 ± 0.13, with no statistical differences between the treatments (Fig. 10d). Similarly, there were no statistical differences in the percentages of CD4^+^ (Fig. 10e) and CD8^+^ (Fig. 10f) between the treatments.

**Figure 11:**
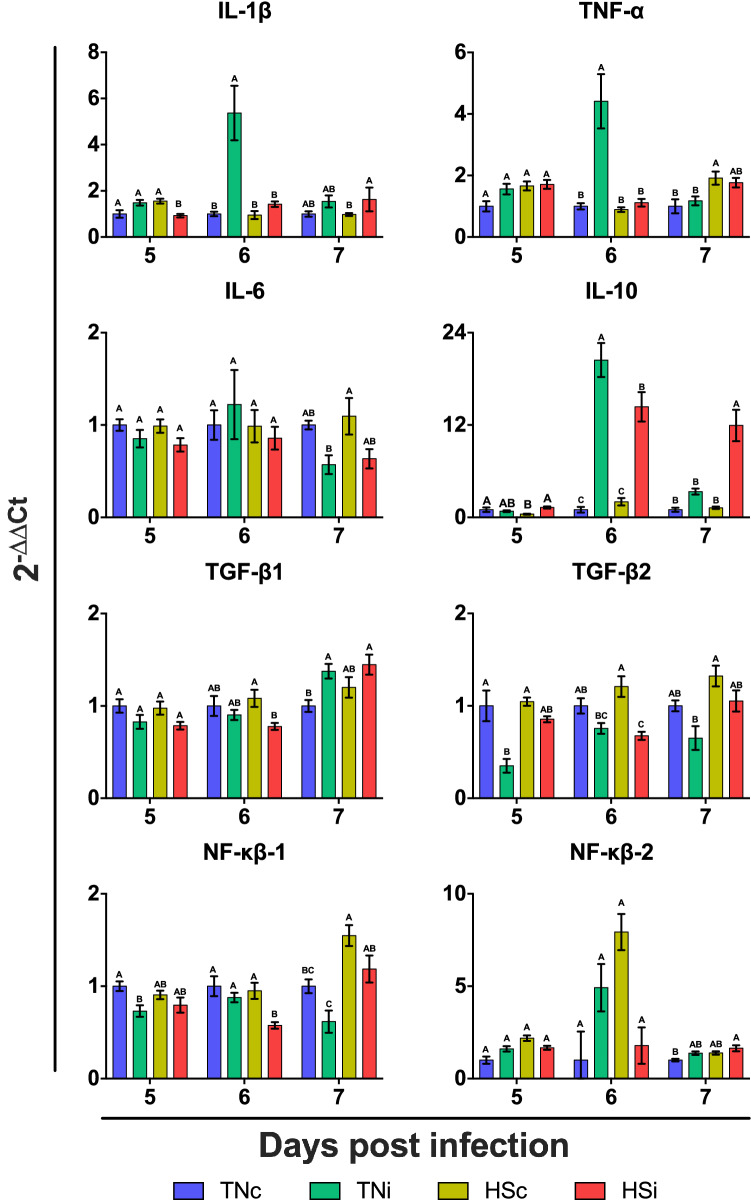
Expression analysis of the cytokine genes interleukin 1 (IL-1), tumor necrosis factor α (TNF-α), interleukin 6 (IL-6), interleukin 10 (IL-10), transforming growth factor β 1 (TGF-β1), transforming growth factor β 2 (TGF-β2), nuclear factor κβ 1 (NF-κβ-1), nuclear factor κβ 2 (NF-κβ-2) chickens infected with *E. maxima* and housed at thermoneutral (TNi; green) or heat stress (HSi; red) environment, as compared to uninfected chickens housed at thermoneutral (TNc; blue) or heat stress (HSc; yellow) environments, assessed from 5 to 7 dpi. Expression of mRNA was done by RT-qPCR, with results expressed as 2^−∆∆Ct^. Data were analyzed by GLM procedure with multiple comparisons corrected with Tukey’s method at 5% level of significance (p < 0.05). Mean and standard error of the mean (SEM) are depicted. Significant differences between the groups are indicated by different superscript letters.

The mRNA expression of host IL-1, TNF-α, IL-6, IL-10, TGF-B1, TGF-B2, NF-κB-1 and NF-κB-2 was assessed at 5 to 7 dpi, and results depicted on Fig. 11. There was a significant downregulation of IL-1 in HSi as compared to TNc, TNi and HSc at 5 dpi, followed by upregulation of IL-1 in TNi at 6 dpi. IL-1 was downregulated in HSc as compared to HSi at 7 dpi, without statistical differences between TNc, TNi and HSC, or between TNc, TNi and HSi. There were no statistical differences in the expression of TNF-α at 5 dpi, however this gene was significantly upregulated in TNi at 6 dpi, and in HSc at 7 dpi. There were no statistical differences in the expression of IL-6 at 5 and 6 dpi between the treatments. At 7 dpi, IL-6 was upregulated in HSc as compared to TNi, however no statistical differences were recorded between TNc, TNi and HSi, or between TNc, HSc and HSi. IL-10 was upregulated in HSi as compared to TNc and HSc at 5 dpi. There was a significant upregulation of IL-10 in TNi at 6 as compared to HSi, TNc and HSc. Similarly, there was a significant upregulation of IL-10 in HSi as compared to the uninfected controls at 6 dpi. The expression of IL-10 remained upregulated in HSi at 7 dpi, however there were no statistical differences in the expression of this gene in TNc, TNi and HSc at 7 dpi. The expression of TGF-β1 was unaltered at 5 dpi, downregulated in HSi at 6 dpi, and upregulated in TNi, and HSi at 7 dpi. The expression of TGF-β2 was downregulated in TNi at 5 and 7 dpi, and in HSi at 6 dpi. The expression of NF-κβ-1 was downregulated in TNi as compared to TNc at 5 dpi, with no statistical differences between TNi, HSc and HSi. There was a significant downregulation of NF-κβ-1 at 6 dpi. NF-κβ-1 was significantly upregulated in HSc at 7 dpi as compared to TNc and TNi. There were no statistical differences in the expression levels of NF-κβ-1 between HSc and HSi, between TNc and HSi, or between TNc and TNi. The expression of NF-κβ-2 was statistically similar between the groups at 5 and 6 dpi. There was a significant upregulation or NF-κβ-2 in HSi at 7 dpi as compared to TNc, but this upregulation was not significant when compared to TNi and HSc.

## Discussion

In poultry, *Eimeria* spp. replicates in the intestine causing extensive tissue damage. *E. maxima* causes congestion and edema, cellular infiltration, thickening of the mucosa, with the later generations of schizonts and sexual stages developing deeper in the tissues, causing considerable disruption of the mucosa^2^, forming a port of entry for secondary infections such as necrotic enteritis^11^. We have previously reported the shortened life cycle of *E. tenella* under HS in vitro and in vivo, as evidenced by the reduction in merozoite replication and consequently oocyst shedding^9^. The objective of this study is to understand some of the underlying mechanisms of the detrimental effect of HS on *Eimeria* spp. and on its interactions with the host.

In the current study, HS was confirmed by physiological response of the animals, as marked by panting and opened wings. Our results demonstrate that, at the level of infection tested, *E*. *maxima* infection in meat-type chickens is as detrimental as HS when infection occurs at 14 days of age, as demonstrated by the similarity in performance parameters evaluated. Between 7 and 14 dpi (14–21 days of age), TNi chickens showed an overall tendency to recover from infection faster, as demonstrated by higher BW, FI and RG as compared to HSi chickens. The recovery from infection coincided with significant reduction in oocyst counts, lesion scores and microscores in TNi as compared to HSi. We also demonstrate that HS by itself exerts severe detrimental effects for poultry production, independent of coccidiosis infection, such as lower development and weight gain. These results are in concordance with literature reports for uninfected heat-stressed broilers, indicating decrease in BW and FI^12^. Although not statistically significant, we also demonstrate that TNi chickens have higher FCR as compared to HSi. Interestingly, our data also reveals that there are no statistical differences in FCR between TNc, HSc and HSi, indicating an effect of heat stress on parasite development and consequently, FCR.

The peak of intestinal lesions was at 6 dpi, and at that day, both TNi and HSi reached similar levels of lesions, indicating that both groups presented significant parasite replication in the intestine. Moreover, at 7 dpi, TNi showed a tendency towards less severe intestinal lesions, as compared to HSi, indicating a faster recover from infection. Following a similar trend, the peak of microscopic scores was at 6 dpi, with a similarity in scores between both infected groups (TNi and HSi). At 7 dpi, microscopic scores tended (non-significant) to be less severe in TNi chickens as compared to its counterpart, HSi. Altogether these data demonstrate that, at the level of infection tested, heat stressed chickens reach similar levels of intestinal damage caused by *E*. *maxima* and are rendered unprepared to overcome infection as fast as chickens raised in a thermoneutral environment.

Intestinal damage was also assessed by disruption of the intestinal blood barrier determined by the concentration of FITC-d in the serum. While both groups of infected chickens reached similar levels of intestinal barrier disruption at 6 dpi, only TNi chickens showed significant disruption of the intestinal blood barrier at 5 dpi, indicating a slower replication of *E*. *maxima* in heat stressed chickens as opposed to the faster replication of the parasite in chickens housed in a thermoneutral environment.

The reduction in oocyst shedding of *E. acervulina*^10^ and *E. tenella*^9^ under conditions has been previously reported. Similarly, HSi chickens also presented a significant reduction in oocyst shedding at 7 dpi as compared to TNi chickens. Parasite replication was further quantified by qRT-PCR. Our results demonstrate that *E*. *maxima* replicate faster in TNi chickens as compared to HSi, indicating that of the host also exerts a detrimental effect on *E*. *maxima*. There is a significant upregulation in 18 s at 7 dpi in HSi chickens, as compared to TNi, indicating that the life cycle of *E*. *maxima* in HS chickens might be in fact extended for a period longer than usual. This delay in parasite development at 7 dpi is also noticeable on histology sections of the small intestine. While moderate to severe coccidiosis was reported for TNi, marked by the presence of several stages of development of *E*. *maxima* in intestinal sections, including macrogametocytes and microgametocytes, only rare asexual stages of *E*. *maxima* were seen in sections from HSi chickens. These are indicatives of the extended asexual development of *E*. *maxima* in HS chickens. The significant increase in expression of 18 s at 7 dpi overlaps with the excretion of *E*. *maxima* oocyst in the feces. Therefore, this increase is most likely an artifact observed due to the excretion of *E*. *maxima* from the intestines of TNi chickens.

We evaluated the development of the parasite with a panel of genes related to oocyst production and macrogametocyte development (GAM56 and GAM82), Two putative genes (HAP-2 and Prot) previously related to microgametocyte development in *E*. t*enella*^13^, and two genes (IMC and EF-2) expressed throughout the development of *E*. *maxima*^14^. Gene expression analysis reveals a significant upregulation of IMC in HSi at 5 dpi and similar expression of EF-2 between HSi and TNi at 5 and 6 dpi, indicating an overall similar level of *E*. *maxima* asexual reproduction in both treatments. Our analysis also reveals significantly lower expression of *Eimeria* spp. genes related to the sexual reproduction (GAM56, GAM82, HAP-2 and Prot) in HSi as compared to TNi.

IMC is a component of the glideosome, the locomotory system believed to be used by *E*. maxima to achieve their characteristic gliding motility^14^. The upregulation of this gene in HSi at 5 dpi indicates higher motility of *E*. *maxima* in the ileum of these chickens as compared to TNi. There was a significantly lower expression of IMC at 6 and 7 dpi, indicating that at those time points, motility of merozoites is compromised in HSi chickens. EF-2 is a common immunodominant antigen^15^ expressed in *E*. *maxima*^14^. EF-2 plays a crucial role in the elongation stage of mRNA translation in eukaryotes, by mediating the translocation of the ribosome relative to the mRNA after addition of each amino acid to the nascent chain^16,17^. The family of proteins is highly conserved and expressed in all eukaryotic cells, playing important functions in signal transduction during mitosis, apoptosis, control of the cell cycle, and utilization of proteins^18,19^. The downregulation of this gene in HSi at 7 dpi strongly indicates reduced *E*. *maxima* replication on this group of chickens. It is important to note, however, that the functions of IMC and EF-2 are putative and have not been yet confirmed at the protein level. The genes GAM56 and GAM82 code for formerly characterized gametocyte antigens and oocyst wall proteins, components of the wall-forming bodies of the macrogametes and integrated into the oocyst wall of *E*. maxima^20–22^. The upregulation of these two genes in TNi agrees with the augmented oocyst production as compared to HSi. HAP-2, a microgametocyte-specific gene previously reported in *E. tenella*^13^ and *Plasmodium*, was also downregulated in parasites from HSi chickens. The expression of this gene is required for gamete fusion and subsequent fertilization of *Plasmodium*^23–25^ and is believed to have similar functions in *E. tenella*, mediating the fusion of the membrane between mating gametes^26^. The downregulation of HAP-2 in HSi indicates that there is a significant reduction in parasite fertilization when hosts are exposed to HS. Prot is a histone-like protein that constrains sperm DNA, resulting in the condensation of the genome into an static state^27^. The gene has been shown to be upregulated in microgametes of *E. tenella*^13^. The downregulation of Prot in the parasite population from HSi chickens is in agreement with the downregulation of the other genes whose expression is specific to the sexual stage of *Eimeria* spp., indicating lower sexual reproduction of *E*. *maxima* in HSi as compared to TNi. At this point, however, the functionality of Prot and HAP-2 in *E*. *maxima* is only putative, based on data from other apicomplexan parasites. Further studies should confirm these genes’ functions at the protein level.

A previous study shows the effect of *Eimeria* spp on the reduction in white blood cell count, antibody production, lymphocytes numbers, spleen weight, macrophage activity; CD4^+^ and CD8^+^ lymphocyte counts^12^. Moreover, Khajavi, et al.^28^ demonstrates that the increase in CD4^+^:CD8^+^ ratio in HS chickens occurs despite decrease in both CD4^+^ and CD8^+^ numbers. Our data demonstrate that the CD4^+^:CD8^+^ ratio increases in chickens upon infection and/or HS, without additive effects. Moreover, HS and *E*. *maxima* infection cause a significant increase in CD4^+^ at 7 dpi, while *E*. *maxima* infection causes a decrease in CD8^+^ cells at 7 dpi but there were no differences in CD4^+^ and CD8^+^ populations between TNi and HSi chickens. Furthermore, our data indicates that the detrimental effects of the host on the outcome of *E. maxima* infection is not due to immunosuppression, as demonstrated by the comparable CD4^+^:CD8^+^ ratios in both groups of infected chickens, TNi and HSi, during the first and second weeks of infection. Further studies should determine whether HS during primary infection limits the host’s immune response to challenge. Due to the systemic effects of HS in the host, we decided to assess the systemic variation in the CD4^+^:CD8^+^ ratio. Future studies should also focus in the intestinal evaluation of T-lymphocytes.

It has been shown that *Eimeria* spp. activates cytokine release and the migration of the inflammatory cells that modulate the host immune system in different ways according to the species^29,30^. Stressors have also been reported to modulate the production and release of cytokines and other inflammatory mediators^31^. To better understand the changes in disease pathogenesis on the current study, we conducted a comprehensive panel analysis of anti-inflammatory and pro-inflammatory cytokines. Heat stress significantly downregulates the expression of pro-inflammatory cytokines (IL-1 and TNF-α) in the ileum of chickens infected with *E*. *maxima*, with the simultaneous downregulation of NF-κβ-1 and upregulation of IL-10. There is the possibility, however, that this diminished cytokine gene expression is a result of the reduced parasite development in HSi chickens. However, it remains unclear at this point the exact mechanism of cytokine response elicited by HS that limits *E*. *maxima* development. There was an upregulation of IL-10 at the peak of intestinal lesions (6 dpi) in TNi and HSi. Interestingly, there is an upregulation of IL-10 only in HSi at 7 dpi, putatively indicating continuation of the replication of this parasite population, corroborating the quantification of *E*. *maxima* population in the ileum.

Results from our previous studies have reported the significant reduction in merozoite replication and oocyst shedding in *E. tenella *in vitro and in vivo. We, herein, also show that there is a significant detrimental effect of HS in the outcome of *E*. *maxima* infection in broiler chickens, as demonstrated by reduced oocyst output post infection. Moreover, the restricted replication of the parasite in HS chickens is related to reduced expression of genes related to gamete fusion, parasite fertilization, and sexual reproduction, resulting in an overall diminished parasite development in chickens reared under HS conditions. Further studies should assess if these changes are translated at the protein level. We also conclude that, at the level of infection tested, *E*. *maxima* induces a systemic downregulation of CD8^+^ lymphocytes, whereas HS and *E*. *maxima* infection induce upregulation of CD4^+^ lymphocytes. Also, a very diverse cytokine response shows indicatives of reduced inflammatory response during concomitant HS and infection, suggesting this to be one of the mechanisms resulting in reduced sexual replication of *E*. *maxima* in heat stressed chickens.

## Methods

All experiments conducted in this study were performed under the Animal Use Proposal (AUP) A2015 04–005 approved by the Animal Care and Use Committee (IACUC) of the University of Georgia.

### Single oocyst cloning

Freshly sporulated *E*. *maxima* oocysts from a North Carolina field strain were counted suspended in PBS at a concentration of one 500 oocysts/mL. Single oocysts were observed in 2 μL droplets under light microscopy and collected using a pipette. Twelve 14 days old-broilers kept in individual disinfected isolators were infected with one oocyst suspended in 200 μL of PBS via gavage. Fecal matter was collected from each isolator at 7 days post infection (dpi) and analyzed for the presence of oocysts by salt flotation. Briefly, one volume of feces was solubilized in 9 volumes of saturated salt solution and the supernatant used to verify for the presence of oocysts in a McMaster chamber. The oocysts recovered from one of the chickens were sporulated as previously reported^32^, and used to infect a second (p2) and third (p3) passages in chickens. The oocysts from the third passage (p3) were later sporulated and used for the experimental infections of chickens. The purity of the *E*. *maxima* clone was verified by PCR following protocol reported by Jenkins, et al.^33^.

### Experimental design

Three hundred 14-days old Ross708 broiler chickens were divided into 30 groups of 10 chickens each and infected via gavage with 2 × 10^5^ *Eimeria maxima* sporulated oocysts suspended in water and housed at two different temperatures: 15 groups were housed at 20 °C (I-20) and 15 groups at 35 °C (I-35). Similarly, another 300 chickens were mock infected with water and housed at 20 °C (C-20) and 35 °C (C-35). All chickens were raised in batteries with wired floor, with ad libitum access to water. Individual body weights and feed consumption were recorded at the day of infection (day 0), at 7 and 14 days post infection (dpi). From 4 to 7 dpi, 10 chickens from each treatment had blood samples collected and were euthanized by cervical dislocation, following with collection of histology samples of liver, spleen and bursa, intestinal content from jejunum, ileum and caeca. Intestinal lesions and the presence of *E*. *maxima* developmental stages (micro-scores) were scored from 4 to 7 dpi. Oocyst shedding was estimated on feces collected from underneath the pens from 4 to 7 dpi and at 14 dpi. Spleen samples collected at zero, 7 and 14 dpi were used to assess T-cell immune response. Quantification of *E*. *maxima* developmental stages in the ileum was assessed by qPCR from 1 to 7 dpi, and by merozoite collection from 2 to 4 dpi.

### Intestinal permeability

Disruption of the intestinal blood barrier was assessed as previously reported^34^. Briefly, FITC-d was gavaged to chickens at 2.2 mg of FITC-d/bird. Two hours post administrations, blood was collected from the jugular vein and stored in tubes protected from the light and kept at room temperature for 3 h to allow clotting, following with centrifugation (1000 g for 15 min) to separate serum. Fluorescence levels of diluted serum (1:1 in PBS) were measured at an excitation wavelength of 485 nm and emission of 528 nm. FITC-d concentration per mL of serum was calculated based on a standard curve. Serum FITC-d levels were compared across infected and non-infected (control) chickens.

### Intestinal lesion scores

*Eimeria maxima* specific lesions were scored daily according to Johnson and Reid^35^. Lesion scores ranged from 0 to 4, where 0 indicates no gross lesion scores, 1 indicates small amounts of orange mucus, 2 represents mid-intestine filled with orange mucus; 3, ballooned thickened intestines with caseous-looking content; and 4 indicates ballooned intestines with blood clots. For microscopic lesion scores (microscores), a 2.5 cm long portion of the jejunum, proximal to the Meckel’s diverticulum was opened and the mucosa scraped with a dissecting knife. The scraping was placed on a slide and covered with a slide. 20 fields were examined at 100 × magnification and scored as follows: 0 (no oocyst present), 1 (1–10 oocysts per field), 2 (11–20 oocysts per field), 3 (21–30 oocysts per field), and 4 (more than 30 oocysts per field).

### Oocyst counts

Pooled fecal samples were homogenized and 5 g of feces were dissolved in 50 mL of saturated salt solution, with 5 replicates per treatment. The solution was filtered in a coarse strainer to remove big particles. 1.5 mL of the filtered homogenized suspension was used to count *E. maxima* oocysts in a Macmaster chamber and expressed in oocysts per gram (OPG).

### Nucleic acid extraction

Tissue samples were stored in liquid nitrogen immediately after collection and at − 80 °C for long term storage. RNA was extracted using trizol. In brief, 100 mg of frozen tissue were homogenized with 1 mL of trizol, followed by phase separation with 0.2 mL of chloroform, homogenization, incubation at room temperature (RT) for 3 min, centrifugation (12,000 rpm, 15 min, 4 °C) and transferring 550  μL of the aqueous phase into a tube for RNA precipitation with 0.5 mL of isopropanol, homogenization and incubation (RT, 10 min), centrifugation (12,000 rpm, 10 min, 4 °C), removal of the supernatant and wash of the pellet with 1 mL of 75% ethanol, centrifugation (12,000 rpm, 5 min, 4 °C), removal of the supernatant, incubation (RT, 10 min), and dissolution of the RNA in 100  μL of RNase free water followed by incubation (55–60 °C, 10 min) and storage of RNA at − 80 °C for downstream applications. RNA was cleaned using RNeasy Mini Kit (Qiagen, Hilden, Germany), following producer`s guidelines, and later reverse-transcribed High Capacity cDNA Reverse Transcription Kit (Thermo Fischer Scientific, Waltham, MA).

### Gene expression, quantification and histology of *Eimeria maxima* in ileum samples

Ileum samples were ground in liquid nitrogen and total RNA was extracted using TRIzon reagent (Invitrogen, Carlsbad, CA), purified with RNeasy Mini Kit (Quiagen, Valencia, CA) and treated with RNase-free DNase (Quiagen, Valencia, CA) according to the manufacturer’s instructions. The RNA was suspended in RNase-DNase-free water and concentration measured on a NanoDrop 2000 Spectophotometer (Thermo Fischer Scientific, Wilmington, DE) and stored at − 80 °C. Two micrograms of total RNA were reverse transcribed with high capacity cDNA Reverse Transcription Kit according to manufacturer’s protocol (Applied Biosystems, Foster City, CA) using a Gradient Mastercycler (Eppendorf, Hauppage, NY) for 10 min at 25 °C, 120 min at 37 °C, five min at 85 °C and final cycle at 4 °C. cDNA samples were stored at -25 °C. Complimentary DNA samples were diluted 1:5 prior to cDNA analysis. Each reaction consisted of 2 μL of diluted cDNA, 0.3 μL of forward primer (10 μM), 0.3 μL of reverse primer (10 μM), 7.4 μL of RNase-DNase-free water and 10 μL of Fast SYBR Green Master Mix (Applied Biosystems, Carlsbald, CA). Primer sequences used for the RT-qPCR assays are listed in Table 1. The RT-qPCR conditions were 95 °C for 20 s, followed by 40 cycles of 95 °C for 3 s and 60 °C for 30 s, with Ct values measured at the end of each cycle. In addition, at the end of the reaction, the melting temperature curve of each RT-qPCR was determined. Each biological sample was runt in triplicates using StepOnePlus (Applied Biosystems, Carlsbald, CA). Relative expression of chicken genes was calculated from the amount of the gene-specific cDNA (target) normalized to the amount of chicken β-actin (endogenous control), using the 2^−∆∆*ct*^ method^36^, in which differential mRNA expression was expressed as treatment groups relative to the control group (TNc). Relative expression of *Eimeria*-specific genes was calculated from the amount of the gene-specific cDNA (target) normalized to the amount of 18 s (endogenous control), in which differential mRNA expression was expressed as treatment groups relative to the TNc group. Relative quantification of *E*. *maxima* in ileum samples was calculated from the amount of 18 s cDNA normalized to the amount of chicken β-actin, in which differential mRNA expression was expressed as treatment groups relative to the TNc group.

**Table 1:** Primers used for gene expression analysis of *Eimeria maxima-*specific genes and chicken cytokines in intestinal tissue.

Target	Primer sequences	NCBI accession number	Species
18 s	F: 5′ CGTAACAAGGTTTCCGTAGGT 3′ R: 5′ CCCGAACGAATCACAACAATATG 3′	AF027724.1	*E*. *maxima*
EF-2	F: 5′ GGCGCTTCAGGAGAGAATTAG 3′ R: 5′ TAGATCTCCTCGGGTTCCATC 3′	GO305837	*E*. *maxima*
GAM 56	F: 5′ TGAATGGAGTGACCGCTATTG 3′ R: 5′ CTGCTGCCCTCAGGTTATG 3′	AY129951.2	*E*. *maxima*
GAM 82	F: 5′ CAGAAGGTACTAACAACCCTGTC 3′ R: 5′ GGGTTGAGGGTTTAGGGTTTAG 3′	AY179510.2	*E*. *maxima*
HAP-2	F: 5′ GAGATGCGTTGGGAGACATAG 3′ R: 5′ CACAGTCCTTCTGCGAGTTT 3′	XM_013481675.1	*E*. *maxima*
IMC-1	F: 5′ GAGACACAGGAGAACATCATTCA 3′ R: 5′ CGGATGACCTCTCAACTTCTTC 3′	GO305988	*E*. *maxima*
Prot	F: 5′ TGCTGCTGCACGAGATTATAG 3′ R: 5′ TTAGGGAGACGAGGTGTATGT 3′	XM_013477344.1	*E*. *maxima*
β-Actin	F: 5′ AGACATCAGGGTGTGATGGTTGGT 3′ R: 5′ TCCCAGTTGGTGACAATACCGTGT 3′	NM_205518.1	*G. gallus*
IL-1β	F: 5′ GTCAACATCGCCACCTACAA 3′ R: 5′ CGGTACATACGAGATGGAAACC 3′	NM_204524.1	*G. gallus*
IL-6	F: 5′ CGTTTATGGAGAAGACCGTGAG 3′ R: 5′ GAGGATTGTGCCCGAACTAAA 3′	NM_204628.1	*G. gallus*
IL-10	F: 5′ AGCTGAGGGTGAAGTTTGAG 3′ R: 5′ AACTCATCCAGCAGTTCAGAG 3′	NM_001004414.2	*G. gallus*
NF-κβ-1	F: 5′ CCTCAACCTCACTTCCTTACTC 3′ R: 5′ CTTCAGTGTCCAGTCCTTTGT 3′	NM_205134.1	*G. gallus*
NF-κβ-2	F: 5′ GACATTGAGGTGCGGTTCTAT 3′ R: 5′ GATGGCGTACTGCTTGTGTA 3′	NM_204413.1	*G. gallus*
TGF-β1	F: 5′ CGCTGTACAACCAACACAAC 3′ R: 5′ CGGCCCACGTAGTAAATGAT 3′	NM_001318456.1	*G. gallus*
TGF-β2	F: 5′ TTCCCTTCCTCCTCTCTCATC 3′ R: 5′ GATACTCTGTACCAGCCCTTTG 3′	NM_204413.1	*G. gallus*
TNF-α	F: 5′ TTACAGGAAGGGCAACTCATC 3′ R: 5′ GCGTTGATGCTCTGAAAGATG 3′	NM_204267.1	*G. gallus*

18 s: 18S ribosomal RNA; EF-2: elongation factor 2; HAP-2: Hapless 2; IMC-1: inner membrane complex 1; MIC-1: microneme protein 1; Prot: protamine; IL-1β: Interleukin 1β; IL-6: Interleukin 6; IL-10: Interleukin 10; NF-κβ-1: nuclear factor κβ 1; NF-κβ-2: nuclear factor κβ 2; TGF-β1: transforming growth factor β 1; TGF-β2: transforming growth factor β 2; TNF-α: tumor necrosis factor α; F: forward primer; R: reverse primer.

Ileum samples were collected in a daily basis, fixed in 10% buffered formalin, trimmed into cassettes, routinely processed and embedded in paraffin. Tissue sections were cut and stained with hematoxylin and eosin (H&E), coverslipped and analyzed by light microscopy.

### Tlymphocytes flow cytometry

Spleen were collected and kept in incomplete (no serum added) cell culture media RPMI 1,640 with L-glutamine (GE Life sciences, Pittsburgh, PA) on ice until processing. Tissues were homogenized and cells separated in a 70 μm Falcon cell strainer (Thermo Fischer Scientific, Waltham, MA). Two mL of the homogenized tissue suspended in cell culture media were laid on top of 2 mL of histopaque 1,077 (Sigma Aldrich, St. Louis, MO) and centrifuged at 1,200 rpm for 10 min at 10 °C without breaks. Peripheral blood mononuclear cells (PMBC) were collected in 2 mL microcentrifuge tubes centrifuged (1,200 rpm, 5 min, 10 °C) and washed with cell culture media two times, followed by centrifugation (1,200 rpm, 5 min, 10 °C). Cells were counted and diluted to a final concentration of 10^6^ cells/mL suspended in flow cytometry buffer (PBS added with 0.5% bovine serum albumin and 0.4% 0.5 M EDTA). In a round-bottom 96 well-plate, 10,000 cells were added per well (10 μL), and incubated for 20 min at 4 °C with 90 μL of CD4-PE (1:300) and CD8-FITC (1:100) conjugated monoclonal antibodies (Southern Biotech, Birmingham, AL) diluted in flow cytometry buffer. Single stained and non-stained controls were included. Post incubation, the plate was centrifuged at 800 rcf for 4 min at 10 °C, followed by removal of the supernatant and fixation of the cells in 200 μL of 2% formaldehyde for 15 min. Post fixation, the plate was centrifuged (800 rcf, 4 min, 10 °C), the fixing solution was removed and the cells were resuspended in 200 μL of flow cytometry buffer. Samples were analyzed by flow cytometry in up to 24 h post fixation. Flow cytometry was performed on a CMM CytoFLEX (Beckman Coulter, Indianapolis, IN) with 5,000 events acquired per sample. Samples were analyzed in 2 channels: FITC emission using a 488-525 nm filter, and PE emission using a 561–585 nm filter. A gate was set to encompass the majority of single cells using the images generated by bright field. The FITC and PE gates were drawn based on the intensity of FITC vs. the intensity of PE in single-stained and non-stained controls. Cell populations in the samples were determined post compensation with single-stained and non-stained controls. The CD4^+^:CD8^+^ ratio was calculated by dividing the number of CD4 + T cells by the number of CD8 + T cells.

### Performance parameters

Body weight (BW), feed intake (FI), feed conversion ratio (FCR) and relative growth (RG) were assessed at 7 and 14 dpi. Feed conversion ratio (FCR) was calculated using the following formula: FCR = FI/BW gain. Relative growth was calculated using the following formula: Relative growth = body weight/initial weight × 100, and results expressed as g/g.

### Statistical analysis

Statistical analysis of BW, FI, FCR, RG, lesion scores, microscores, FITC-d and OPG was conducted in Prism 7.0 software (Graphpad Software Inc., San Diego, California). D’Agostino and Pearson test was applied to determine normality and determine whether data should be analyzed by parametric or non-parametric tests. Kruskal–Wallis test with multiple comparisons corrected using Dunn’s method was used to compare non-parametric data. Parametric data were analyzed by two-way-ANOVA test with multiple comparison’s corrected by Tukey’s method. Gene expression and lymphocyte population analysis was conducted in SAS software (SAS Institute, Cary, North Carolina) by GLM procedure with multiple comparisons corrected with Tukey’s method. All tests were performed at the 5% level of significance. All results are expressed as mean ± standard error of the mean.
